# Supplementary material for: Performance of novel onchocerciasis rapid diagnostic tests in a field setting in South Sudan
Source: PLoS Negl Trop Dis. 2025 Nov 21;19(11):e0013736. doi: 10.1371/journal.pntd.0013736 (PMC12637992; doi:10.1371/journal.pntd.0013736)
Supplement: Attachment — Submitted filename: Reviewers Comment PLOS NTD.docx [file pntd.0013736.s002.docx]

Reviewers Comment

Introduction: The introduction was excellently expressed.

Methodology:

i. The coordinate of Mardi county needs to be inserted in line 1 or a reference is made based on previous study
ii. There is need to provide information on the skills of the healthcare workers (HCWs) and if the researchers also provided minimal training or refresher skill before the commencement of the study

Results: Excellently articulated

Discussion and limitations : Well explained

Overall comment: This is an excellent study and would be of interest to larger audience in Oncho community
