# Supplementary material for: Performance of novel onchocerciasis rapid diagnostic tests in a field setting in South Sudan
Source: PLoS Negl Trop Dis. 2025 Nov 21;19(11):e0013736. doi: 10.1371/journal.pntd.0013736 (PMC12637992; doi:10.1371/journal.pntd.0013736)
Supplement: Attachment — Submitted filename: 15.10.25Response to the comments.docx [file pntd.0013736.s003.docx]

Dear editors and reviewers,

Comments in Manuscript:

- Relay > Rely : **Done**
- This reviewer understands the point made by authors, but wonders if the brief period of pregnancy and parturition typically averaging 18-24 months meet the definition of ivermectin naivety in the context of onchocerciasis control, given there are population in hypoendemic region that are more commonly described by this term. May be if a time frame be included so that readers will have better clarity that individuals in this population might have potentially taken ivermectin in the last 2-3 years: **Text changed to “making them one of the few remaining groups unaffected by recent ivermectin exposure.”**
- 10 millilitres > 10 microliters: **Done**
- Authors should specify what 500ul of solution/reagents was added? Was this also in a 96-well plate, what type?: **Text added “500µl of saline within a 96-well plate”**
- Can authors include the information on the positive control used for this assay: **Text added “Each plate included a historical microscopy-positive sample as positive control.”**
- Also, the DNA extraction process described in the methodology didn't use the two biopsy nor centrifuge the saline solution to concentrating the residual, which is the usual standard practice for DNA extraction of this type of sample. This could have impacted the parasite DNA available for detection. This reviewer suggest authors should incorporate this into the limitation/discussion.: **Text added: “, the use of only one skin snip for DNA extraction,”**

 Response to journal requirements:

- We do not publish any copyright or trademark symbols that usually accompany proprietary names, eg ©,  ®, or TM  (e.g. next to drug or reagent names). Therefore please remove all instances of trademark/copyright symbols throughout the text, including:® on pages: 5, and 6. **DONE**
- We note that your Data Availability Statement is currently as follows: "The datasets generated during and/or analysed during the current study are freely available to other researchers.". Please confirm at this time whether or not your submission contains all raw data required to replicate the results of your study. Authors must share the “minimal data set” for their submission. PLOS defines the minimal data set to consist of the data required to replicate all study findings reported in the article, as well as related metadata and methods ([https://journals.plos.org/plosone/s/data-availability#loc-minimal-data-set-definition](https://eur01.safelinks.protection.outlook.com/?url=https%3A%2F%2Fjournals.plos.org%2Fplosone%2Fs%2Fdata-availability%23loc-minimal-data-set-definition&data=05%7C02%7Camber.hadermann%40uantwerpen.be%7C430f32f79cdd42450d5208de00b9e003%7C792e08fb2d544a8eaf72202548136ef6%7C0%7C0%7C638948996498891994%7CUnknown%7CTWFpbGZsb3d8eyJFbXB0eU1hcGkiOnRydWUsIlYiOiIwLjAuMDAwMCIsIlAiOiJXaW4zMiIsIkFOIjoiTWFpbCIsIldUIjoyfQ%3D%3D%7C0%7C%7C%7C&sdata=JgaEhOHn5Ag8U1kw0ooQ2PrN8Q4m6oyyq16Jpyk8ZvM%3D&reserved=0)).
  For example, authors should submit the following data: 
  1) The values behind the means, standard deviations and other measures reported;
  2) The values used to build graphs;
  3) The points extracted from images for analysis..
  Authors do not need to submit their entire data set if only a portion of the data was used in the reported study.
  If your submission does not contain these data, please either upload them as Supporting Information files or deposit them to a stable, public repository and provide us with the relevant URLs, DOIs, or accession numbers. For a list of recommended repositories, please see [https://journals.plos.org/plosone/s/recommended-repositories](https://eur01.safelinks.protection.outlook.com/?url=https%3A%2F%2Fjournals.plos.org%2Fplosone%2Fs%2Frecommended-repositories&data=05%7C02%7Camber.hadermann%40uantwerpen.be%7C430f32f79cdd42450d5208de00b9e003%7C792e08fb2d544a8eaf72202548136ef6%7C0%7C0%7C638948996498909343%7CUnknown%7CTWFpbGZsb3d8eyJFbXB0eU1hcGkiOnRydWUsIlYiOiIwLjAuMDAwMCIsIlAiOiJXaW4zMiIsIkFOIjoiTWFpbCIsIldUIjoyfQ%3D%3D%7C0%7C%7C%7C&sdata=oe%2BIQUpAHYbVXhDS6QbK08z2X1nnQN1SZs3ZnMnj2ns%3D&reserved=0). 
  If there are ethical or legal restrictions on sharing a de-identified data set, please explain them in detail (e.g., data contain potentially sensitive information, data are owned by a third-party organization, etc.) and who has imposed them (e.g., an ethics committee). Please also provide contact information for a data access committee, ethics committee, or other institutional body to which data requests may be sent. If data are owned by a third party, please indicate how others may request data access.

**We hereby confirm that the data sets include all data needed to reproduce the study, and include enough information to understand each variable.**

- Please amend your detailed Financial Disclosure statement. This is published with the article. It must therefore be completed in full sentences and contain the exact wording you wish to be published.
  1) State what role the funders took in the study. If the funders had no role in your study, please state: "The funders had no role in study design, data collection and analysis, decision to publish, or preparation of the manuscript."
  2) If any authors received a salary from any of your funders, please state which authors and which funders..
  If you did not receive any funding for this study, please simply state: u201cThe authors received no specific funding for this work.u201d

**We hereby confirm that the financial disclosure statement includes the necessary information.**

- Kindly revise your competing statement to align with the journal's style guidelines: 'The authors declare that there are no competing interests.' **DONE**

Additional reviewers' comments:

- Methodology:
  - The coordinate of Mardi county needs to be inserted in line 1 or a reference is made based on previous study: **We have added the coordinates of the Maridi hospital within “Sample collection”.**
  - There is need to provide information on the skills of the healthcare workers (HCWs) and if the researchers also provided minimal training or refresher skill before the commencement of the study: **Text included: “All HCW received training for all relevant procedures during two sit-down meetings, which covered standard operation procedures for sample collection and diagnostic tests, and ethical consent. Additional personal one-on-one training sessions were provided as needed.”**

Based on additional comment (included below), no additional changes need to be made.

**Methods**

-Are the objectives of the study clearly articulated with a clear testable hypothesis stated?

-Is the study design appropriate to address the stated objectives?

-Is the population clearly described and appropriate for the hypothesis being tested?

-Is the sample size sufficient to ensure adequate power to address the hypothesis being tested?

-Were correct statistical analysis used to support conclusions?

-Are there concerns about ethical or regulatory requirements being met?

Reviewer #1: Yes.

Reviewer #2: Excellent with minor modification

**Results**


-Does the analysis presented match the analysis plan?

-Are the results clearly and completely presented?

-Are the figures (Tables, Images) of sufficient quality for clarity?

Reviewer #1: (No Response)

Reviewer #2: Excellent

**Conclusions**


-Are the conclusions supported by the data presented?

-Are the limitations of analysis clearly described?

-Do the authors discuss how these data can be helpful to advance our understanding of the topic under study?

-Is public health relevance addressed?

Reviewer #1: The conclusions are supported by data presented

Reviewer #2: Excellent

**Editorial and Data Presentation Modifications?**


Use this section for editorial suggestions as well as relatively minor modifications of existing data that would enhance clarity. If the only modifications needed are minor and/or editorial, you may wish to recommend “Minor Revision” or “Accept”.

Reviewer #1: None

Reviewer #2: None

**Summary and General Comments**


Use this section to provide overall comments, discuss strengths/weaknesses of the study, novelty, significance, general execution and scholarship. You may also include additional comments for the author, including concerns about dual publication, research ethics, or publication ethics. If requesting major revision, please articulate the new experiments that are needed.

Reviewer #1: The design of the study, the presentation of results and overall writing of the manuscripts are acceptable for publication. This is a well designed study that provides useful information to discussion on onchocerciasis diagnosis.

Reviewer #2: Recommended with minor review

PLOS authors have the option to publish the peer review history of their article ([what does this mean?](https://eur01.safelinks.protection.outlook.com/?url=https%3A%2F%2Ftrack.editorialmanager.com%2FCL0%2Fhttps%3A%252F%252Fjournals.plos.org%252Fplosntds%252Fs%252Feditorial-and-peer-review-process%2523loc-peer-review-history%2F1%2F010f01992cce6603-b4fe6442-8eec-4a52-b406-f0506d0323da-000000%2FiB2HoPEwYjcyGmuZ2VvbhhDZA_JOqnAo0hzVrmoZLhE%3D227&data=05%7C02%7Camber.hadermann%40uantwerpen.be%7C430f32f79cdd42450d5208de00b9e003%7C792e08fb2d544a8eaf72202548136ef6%7C0%7C0%7C638948996498926178%7CUnknown%7CTWFpbGZsb3d8eyJFbXB0eU1hcGkiOnRydWUsIlYiOiIwLjAuMDAwMCIsIlAiOiJXaW4zMiIsIkFOIjoiTWFpbCIsIldUIjoyfQ%3D%3D%7C0%7C%7C%7C&sdata=pAhYOdAMs%2FRrlckSi1%2FXd%2BOJ1Lgp93HraRm%2F6pL8joc%3D&reserved=0)). If published, this will include your full peer review and any attached files.


If you choose “no”, your identity will remain anonymous but your review may still be made public.


**Do you want your identity to be public for this peer review?** For information about this choice, including consent withdrawal, please see our [Privacy Policy](https://eur01.safelinks.protection.outlook.com/?url=https%3A%2F%2Ftrack.editorialmanager.com%2FCL0%2Fhttps%3A%252F%252Fwww.plos.org%252Fprivacy-policy%2F1%2F010f01992cce6603-b4fe6442-8eec-4a52-b406-f0506d0323da-000000%2F3Ep26a0hCuLod-cuK-T6MldV4esFcekLUZa6mcKxpws%3D227&data=05%7C02%7Camber.hadermann%40uantwerpen.be%7C430f32f79cdd42450d5208de00b9e003%7C792e08fb2d544a8eaf72202548136ef6%7C0%7C0%7C638948996498943608%7CUnknown%7CTWFpbGZsb3d8eyJFbXB0eU1hcGkiOnRydWUsIlYiOiIwLjAuMDAwMCIsIlAiOiJXaW4zMiIsIkFOIjoiTWFpbCIsIldUIjoyfQ%3D%3D%7C0%7C%7C%7C&sdata=FYvwo3l91qokFi2VCCSS19OWP8vczh9iuE0vFU7ymgE%3D&reserved=0).

Reviewer #1: **Yes:** Adebiyi A Adeniran

Reviewer #2: No

**Response to Editorial comments**

1) We note that your **Data Availability** Statement is currently as follows: "The datasets generated during and/or analysed during the current study are freely available to other researchers.". Please confirm at this time whether or not your submission contains all raw data required to replicate the results of your study. Authors must share the “minimal data set” for their submission. PLOS defines the minimal data set to consist of the data required to replicate all study findings reported in the article, as well as related metadata and methods ([https://journals.plos.org/plosone/s/data-availability#loc-minimal-data-set-definition](https://url.uk.m.mimecastprotect.com/s/0TxZCAMzlil4B6LtQt1uGSNdX?domain=journals.plos.org)).
   For example, authors should submit the following data:
   1) The values behind the means, standard deviations and other measures reported;
   2) The values used to build graphs;
   3) The points extracted from images for analysis..
   Authors do not need to submit their entire data set if only a portion of the data was used in the reported study.
   If your submission does not contain these data, please either upload them as Supporting Information files or deposit them to a stable, public repository and provide us with the relevant URLs, DOIs, or accession numbers. For a list of recommended repositories, please see [https://journals.plos.org/plosone/s/recommended-repositories](https://url.uk.m.mimecastprotect.com/s/cS3UCBPAmURj06ph1u3u2wKj8?domain=journals.plos.org).
   If there are ethical or legal restrictions on sharing a de-identified data set, please explain them in detail (e.g., data contain potentially sensitive information, data are owned by a third-party organization, etc.) and who has imposed them (e.g., an ethics committee). Please also provide contact information for a data access committee, ethics committee, or other institutional body to which data requests may be sent. If data are owned by a third party, please indicate how others may request data access.

**Response**

**We now mention in the paper “The datasets generated during and/or analysed during the current study are publicly available in the Zenedo repository (DOI: 10.5281/zenodo.17357017).”**

2) Please amend your detailed Financial Disclosure statement. This is published with the article. It must therefore be completed in full sentences and contain the exact wording you wish to be published.
1) State the initials, alongside each funding source, of each author to receive each grant. For example: "This work was supported by the National Institutes of Health (####### to AM; ###### to CJ) and the National Science Foundation (###### to AM)."
2) State what role the funders took in the study. If the funders had no role in your study, please state: "The funders had no role in study design, data collection and analysis, decision to publish, or preparation of the manuscript.".
If you did not receive any funding for this study, please simply state: u201cThe authors received no specific funding for this work.u201d

**Response**

**We now state “This work was supported by the Coalition for Operational Research on Neglected Tropical Diseases (COR-NTD), which is funded at The Task Force for Global Health primarily by the Bill & Melinda Gates Foundation (OPP1190754) and by the United States	Agency for International Development (USAID) (COR-NTD grant to RC). The study funders had no role in study design, data collection and analysis, decision to publish or preparation of the manuscript.”**

3) We have amended your Competing Interest statement to comply with journal style. We kindly ask that you double check the statement and let us know if anything is incorrect.

**Response**

**The Competing Interest statement is correct**
